# Ebola Outbreak in Nigeria: Increasing Ebola Knowledge of Volunteer Health Advisors

**DOI:** 10.5539/gjhs.v8n1p72

**Published:** 2015-05-15

**Authors:** Unnati Patel, Jennifer R. Pharr, Chidi Ihesiaba, Frances U. Oduenyi, Aaron T. Hunt, Dina Patel, Michael Obiefune, Nkem Chukwumerije, Echezona E. Ezeanolue

**Affiliations:** 1 HealthySunrise Foundation, Las Vegas, NV, USA; 2 Global Solutions for Prevention, education, Treatment, Training and Research (PeTR-GS), Enugu, Nigeria; 3 University of Nevada School of Medicine, Las Vegas, NV, USA; 4 University of Nevada, Las Vegas, Las Vegas, NV, USA; 5 Association of Nigeria Physicians in the Americas (ANPA), Durham, NC, USA

**Keywords:** Ebola virus disease, low income countries, volunteer health advisors

## Abstract

In many low-income countries, volunteer health advisors (VHAs) play an important role in disseminating information, especially in rural or hard-to-reach locations. When the world’s largest outbreak of Ebola virus disease (EVD) occurred in 2014, a majority of cases were concentrated in the West African countries of Guinea, Liberia, and Sierra Leone. Twenty cases were reported in Nigeria initially and there was a need to rapidly disseminate factual information on Ebola virus. In southeast Nigeria, a group of VHAs was being used to implement the Healthy Beginning Initiative [HBI], a congregation based intervention to increase HIV testing among pregnant women and their male partners. The purpose of this study was to assess the baseline and post EVD training knowledge of VHAs during the outbreak in Nigeria. In September 2014, 59 VHAs attending a HBI training workshop in the Enugu State of Nigeria participated in an Ebola awareness training session. Participants completed a 10-item single-answer questionnaire that assessed knowledge of Ebola epidemiology, symptoms, transmission, prevention practices, treatment and survival prior to the Ebola awareness training. After the training, the VHAs repeated the questionnaire. Answers to pre and post questionnaires were analyzed using paired t-tests. Multiple linear regression was used to examine the relationship between pre and post total questionnaire scores and age, education, current location and employment. The average pre-test score was 7.3 and average post-test score was 7.8 which was a significant difference (t=-2.5, p=0.01). Prior to the training, there was a significant difference in Ebola knowledge based on the VHAs education only (p<0.01). After training, education was no longer significant for Ebola knowledge. Existing community health programs can be used as a platform to train VHAs in times of epidemics for quick dissemination of vital health information in areas lacking adequate health infrastructure and personnel.

## 1. Introduction

### 1.1 Ebola Virus Disease (EVD) Outbreak

In the world’s largest outbreak of Ebola virus disease (EVD), there had been 20,206 cases, with 7,905 deaths as of December 31, 2014 (World Health Organization, 2015). The epidemic began in December of 2013 in Guinea. Nine months after the first case of Ebola was reported, confirmed cases and deaths were reported in the five West African countries of Guinea, Liberia, Sierra Leone, Senegal and Nigeria; however, the majority of cases were concentrated in Guinea, Liberia, and Sierra Leone (Team, 2014). Analysis of this outbreak by the World Health Organization (WHO) found that, although it was unprecedented in size, it was very similar to other Ebola epidemics in terms of incubation period, duration of illness, case fatality rate and reproduction number (Team, 2014). Over 60% of the people with EVD were between the ages of 15 and 44 with an equal distribution of EVD between men and women (Team, 2014). Symptoms were consistent with other Ebola epidemics and included: fever, fatigue, loss of appetite, vomiting, diarrhea, headache, and abdominal pain. In September of 2014, the case fatality rate was calculated at 70.8% which was consistent between Guinea, Liberia, and Sierra Leone (Team, 2014).

The first case to reach Nigeria occurred on July 20, 2014 when a patient hospitalized in Liberia flew to Lagos against medical advice. The patient was symptomatic enroute and ill at the time of landing in Lagos, Nigeria (World Health Organization, 2014). Once EVD was confirmed in this patient, Nigeria’s Federal Ministry of Health declared an Ebola emergency and opened the Emergency Operations Center. The center initiated an Incident Management System to centralize the Ebola response in the country (Shuaib et al., 2014). First, through epidemiological investigation, a team of 150 “contact tracers” tracked down and identified 898 contacts linked to the index case. These contacts were under surveillance and had a total of 18,500 face-to-face visits to check for fever and other symptoms of EVD. Symptomatic individuals were swiftly isolated for further testing. “Social mobilizers” targeted areas surrounding EVD contacts to disperse health information and dispel false rumors, reaching about 26,000 households in total (Fasina et al., 2014). With this organized and aggressive, centralized system, Nigeria had a lower case fatality rate (45.5%) than Guinea, Liberia, and Sierra Leone and was officially declared Ebola-free on October 19, 2014. In total, 20 EVD cases were reported in Nigeria (19 laboratory confirmed, 1 suspected) between July 20 and August 31, with no new cases afterwards (World Health Organization, 2014).

During the outbreak, the spread of misinformation in Nigeria was evident on the ground and online. The most common rumors spread online were about drinking or washing with salt water or that EVD could be cured with the ewedu plant or by unqualified blood transfusion (Oyeyemi, Gabarron, & Wynn, 2014). Mistrust of health-care workers and the government, along with rumors of EVD being a Western-designed propaganda were also present (Yusuf, Ahmad, & Yee, 2014). During an outbreak of a highly infectious disease such as Ebola, it is important to be able to quickly disseminate information about symptoms, transmission, prevention practices and treatment. In many low-income countries with an insufficient number of trained health professionals (doctors and nurses), community or voluntary health workers play an important role in disseminating information, especially in rural or hard-to-reach locations (Lehmann & Sanders, 2007; Perry, Zulliger, & Rogers, 2014). This was evident in the 2000 outbreak of Ebola in Uganda. Community mobilization for timely information dissemination using selected village volunteers was critical in controlling the epidemic which lasted for 42 days and resulted in 224 deaths (Okware et al., 2002).

### 1.2 Use of Community Health Workers for Information Dissemination

Community health worker (CHW) is an umbrella term used for a variety of health aides who work within their communities. Other terms include: lay health worker, volunteer and voluntary health worker or health promoter (Lehmann & Sanders, 2007). CHWs are as varied as the communities they serve and can be “men or women, young or old, literate or illiterate” (Lehmann & Sanders, 2007). Research has shown that community health workers can be very effective in reducing the burden of disease, particularly in low-income countries (Perry, Zulliger, & Rogers, 2014). CHWs have helped improve childhood nutrition by promoting breast feeding and nutrition support for undernourished infants and children, reduced neonatal mortality by providing mothers with education and promoting sanitation and hygiene, and reduced childhood mortality by teaching mothers how to treat diarrhea in their children (Perry et al., 2014). Additionally, CHWs have helped reduce the spread of HIV/AIDS by providing support to caregivers, educating the population about HIV prevention and providing HIV testing and counseling. CHWs have also helped control other infectious diseases such as malaria and tuberculosis through education about prevention and diagnosis (Perry et al., 2014).

### 1.3 The Healthy Beginning Initiative in Nigeria

Nearly 90% of people in Nigeria report going to a worship center (church or mosque) at least once per week, and Nigeria has an extensive network of faith-based institutions (World Values Survey, 2015). Religious leaders understand that they can use their position for infectious disease prevention and interventions (Ucheaga & Hartwig, 2010). Building on this background, the Healthy Beginning Initiative (HBI) was developed. HBI is a culturally adapted, family-centered approach that relies on the wide distribution of churches and church-based community networks to improve maternal and child health. In the Baby Shower Trial, a total of 40 churches in Enugu State of Nigeria were invited to participate in the HBI. Each church has a Health Team (HT) comprised of the priest, women’s and men’s leaders and two volunteer health advisors (VHAs), one of whom is trained as a phlebotomist (Ezeanolue et al., 2013). The VHAs are lay, trusted community members who volunteer their time to be trained by the program and act as their community source for health information. HBI promotes individual testing for diseases such as HIV, malaria, sickle cell, hepatitis, and syphilis and provides education about prevention of infectious diseases (HIV, hepatitis, syphilis and malaria). Additionally, the program helps with tracking and retention of participants who are found to have one of these diseases (Ezeanolue et al., 2013).

The HBI became a well-established program and has been successful in recruiting members of the congregation to participate in various health related activities (Ezeanolue et al., 2013). The HBI has already shown promising results in a study regarding HIV testing among pregnant women and their male partners. HBI has also been shown to be effective for mental health screenings among participants (Iheanacho et al., 2014). In a study, 93% of the HBI participants agreed to complete a mental health questionnaire. Twenty-one percent of the participants were found to have significant psychological distress. The conclusion of this study was that VHAs could be trained to administer mental health screenings (Iheanacho et al., 2014).

### 1.4 Purpose of Study

The purpose of this study was to assess the baseline and post-intervention EVD knowledge of VHAs participating in the HBI during the EVD outbreak in Nigeria. Additionally, we sought to determine the feasibility of using existing systems such as a church-based community health program to quickly bring together, assess, educate and train VHAs about EVD prior to returning to their respective communities in rural, hard-to-reach communities.

## 2. Methods

### 2.1 Setting and Population

On September 25^th^ and 26^th^ of 2014, sixty of the eighty VHAs attended a two-day HBI update and training workshop in the Enugu State of Nigeria. In addition to receiving information about the HBI program, participates were asked to participate in an Ebola awareness training session. Fifty-nine of the sixty VHAs agreed to participate.

### 2.2 Data Collection

Participants completed a 10-item single-answer questionnaire that assessed knowledge of Ebola epidemiology, symptoms, transmission, prevention practices, treatment and survival prior to an Ebola awareness training. The Ebola awareness training was produced by SOS International and used with their permission. The day after the training, the VHAs completed the questionnaire again (post-training). Answers to the questions pre and post-training were analyzed and reviewed with participants who subsequently scheduled Ebola awareness presentations at their congregations. This study was approved by the University of Nevada, Reno IRB.

### 2.3 Data Analysis

All analyses were performed using SPSS 21. Descriptive characteristics of the sample were produced. Answers to pre and post-training questionnaires were analyzed using paired t-tests. Multiple linear regression was used to examine the relationship between pre and post total questionnaire scores (dependent variable) and age, education, current location and employment. Statistical analysis was accomplished using a 5% significance level.

## 3. Results

### 3.1 Characteristics of the Sample

Fifty-seven of the 60 VHA in attendance during the 2-day training completed both the pre and post questionnaire (Response Rate=95%). Participants mean age was 41.2 years. A majority of participants were females (77%), resided in rural areas (58%), were college-educated (61%), employed either full or part-time (73%) and married (80%) (Table 1).

**Table 1:** Demographic Characteristics of VHAs

	Mean	Standard Deviation
**Age**		
	41.2	10.5

	**Frequency**	**Percent**

**Gender**		
Missing	2	3.5
Female	44	77.2
Male	11	19.3

**Education**		
Missing	2	3.5
Primary 6th	5	8.8
Secondary 12th	15	26.3
Tertiary	35	61.4

**Current Residence**		
Missing	4	7.0
Rural	33	57.9
Semi-Urban	5	8.8
Urban	15	26.3

**Religion**		
Missing	2	3.5
Anglican	15	26.3
Catholic	39	68.4
Other	1	1.8

**Employment**		
Missing	4	7.0
Full-Time	33	57.9
Part-Time	9	15.8
Unemployed	11	19.3

**Marital Status**		
Missing	2	3.5
Married	46	80.7
Single	7	12.3
Widowed	2	3.5

### 3.2 Ebola Knowledge

The average pre-test score for participants was 7.3 and average post-test score was 7.8 out of ten. This difference was significant (t=-2.5, p=0.01) when analyzed using a paired t-test. Prior to the training, there was a significant difference in Ebola knowledge (total questionnaire score) based on the VHAs education only (p<0.01). Tertiary education was used as the reference and VHAs with tertiary education had significantly higher knowledge scores compared to VHAs with primary (p<0.01) or secondary (p=0.03) education. Age, current location and employment were not significant. After training, education was no longer significant for Ebola knowledge.

The percentages of correct responses pre/post-training are provided in Table 2. Prior to the training, six participants got less than fifty percent of the questions correct. After the training, three of the VHAs scored less than five out of ten. Only 45% of participants knew the correct duration before symptoms could be seen in an infected patient prior to training which increased to 88% after training. A majority of participants correctly identified hand washing as the best way to prevent Ebola (93% pre and 100% post) and knew that dead bodies could still be infected (83% pre and 84% post). Additionally, a high percentage of VHAs knew that a patient with symptoms of Ebola should go to the nearest hospital (88% pre and 93% post). Prior to training, 53% believed there was an Ebola vaccine which decreased to 30% post training. However, prior to training, 49% of the VHAs thought most people infected with EVD survived which increased to 74% post training and is incorrect.

**Table 2:** Percentage of Correct Answers to the Ebola Knowledge Questionnaire, Pre and Post Training

Question	Pre	Post
1. Ebola virus is only found in Humans True/False	82.5	73.7
2. Ebola Virus is not found in the Air? True/False	77.2	73.7
3. Early symptoms of Ebola Virus include: a. Fever b. Tiredness c. Headaches d. Nausea e. All of the above	75.4	75.4
4. You may notice symptoms within 2 days of coming in contact with someone infected with Ebola virus. True/False	45.6	87.7
5. Late symptoms of Ebola Virus include: a. Vomiting b. Diarrhea c. Cough d. Bleeding e. All of the above	71.9	71.9
6. Ebola can be treated by vaccine True/False	47.4	70.2
7. Dead bodies can no longer infect other people with Ebola Virus True/False	82.5	84.2
8. Most people with Ebola virus survive True/False	50.9	26.3
9. Best way to prevent Ebola is to wash your hands often with Soap True/False	93.0	100.0
10. You should go to the nearest hospital if you feel you are infected with the Ebola Virus True/False	87.7	93.0

## 4. Discussion

This study focused on determining the EVD baseline knowledge of VHAs in rural Nigeria and their knowledge after training. The pre-training survey showed that VHAs had adequate knowledge about EVD; however, gaps in knowledge were identified. By reviewing answers and conducting a short training session, VHAs knowledge of Ebola was significantly increased. During the training, they were also provided with strategies for information dissemination. This along with increased knowledge could prepare them for rapid information dissemination in their rural and hard-to-reach communities; however, their ability to disseminate the information was not measured. Prior to training, there was a significant difference in Ebola knowledge based on level of education. After training, this difference was negated. The VHAs did not have a medical background and most of the incorrect responses were medically-specific questions. We did see a decrease in correct responses to some of the questions which may have resulted from the training provided. Because we only talked about EVD in humans, there was a decrease in the number of correct responses to the statement that ‘Ebola is only found in humans’. Additionally, because we focused on the importance of accessing medical care at the first signs and symptoms of EVD, the VHAs may have perceived that a person with EVD would survive with prompt medical care. This may have caused the decrease in the number of correct responses to the statement that most people survive Ebola. The majority of VHAs answered important public health questions correctly, specifically questions about symptoms, transmission, prevention practices and treatment.

Results from this study also demonstrate that by using existing systems such as a church-based community health program, VHAs from the HBI could be quickly brought together, assessed, educated and trained on EVD knowledge prior to returning to their respective communities. By using an existing system, time and money were saved, which is of vital importance in infectious disease epidemics. The project was based in rural southeastern Nigeria, where the healthcare infrastructure is not well developed compared to urban centers of Nigeria (Ademiluyi & Aluko-Arowolo, 2009). This leaves a gap in medical care in rural areas. VHAs can fill this gap by utilizing existing infrastructure like a church-based community health program to reach a larger population of Nigerians who may not have access to relevant EVD knowledge, especially when timely dissemination of information is critical (Okware et al., 2002).

While most of the literature pertaining to CHWs is concentrated around maternal-child health, chronic health care or more prevalent infectious diseases such as HIV/AIDS, TB, and malaria, there is a dearth of information about their role in times of epidemics such as the EVD outbreak in 2014 (Lehmann & Sanders, 2007; Perry et al., 2014). This study showed that CHWs may contribute to rapid responses in time-sensitive epidemics and reach less developed and poorer areas. In future epidemics, it may prove beneficial to employ CHWs to assist in preventing the rapid spread of disease.

## 5. Conclusion

Findings from this study indicate that VHAs with no medical background had adequate baseline EVD knowledge and that a training session to educate the VHAs was effective in significantly increasing their EVD knowledge. Existing community health programs such as the HBI can be used as a platform to quickly train VHAs in times of epidemics with the potential to disseminate vital health information in areas lacking adequate health infrastructure.
